# Genomic and Phenotypic Insights into Carbapenem-Resistant *Pseudomonas aeruginosa* in the Aquatic Environments of the Tibetan Plateau

**DOI:** 10.3390/microorganisms14051094

**Published:** 2026-05-12

**Authors:** Dingxiang Lu, Lin Liu, Zhongwei Yang, Tianjiao Chen, Dong Yang, Danyang Shi, Shuqing Zhou, Junwen Li, Haibei Li, Min Jin

**Affiliations:** Tianjin Key Laboratory of Risk Assessment and Control for Environment & Food Safety, State Key Laboratory of Pathogen and Biosecurity, Academy of Military Medical Sciences, Tianjin 300050, China; dingxiang9920@163.com (D.L.); liulinhaiyi@163.com (L.L.); tju_yzw1986@foxmail.com (Z.Y.); c2323ctjann@163.com (T.C.); yangd8611@163.com (D.Y.); shidanyangsdy@163.com (D.S.); zsq13821851449@163.com (S.Z.); junwen9999@hotmail.com (J.L.)

**Keywords:** carbapenem-resistant *Pseudomonas aeruginosa*, Tibetan Plateau, aquatic environments, antibiotic resistance, virulence

## Abstract

Carbapenem-resistant *Pseudomonas aeruginosa* is increasingly becoming a global health threat. However, although aquatic environments are key resistance reservoirs, data obtained from high-altitude ecosystems are scarce. Whole-genome sequencing of eight carbapenem-resistant *P. aeruginosa* isolates collected from aquatic environments in the Tibetan Plateau identified three sequence types (STs), with ST1420 predominating (62.5%, 5/8). Phylogenetic analysis revealed a close clustering of isolates with those from distant clinical settings, suggesting potential cross-habitat transmission. All studied strains were multidrug-resistant, exhibiting 100% resistance to imipenem, ceftriaxone, and trimethoprim–sulfamethoxazole. This included the PA6 strain, which showed multiple-antibiotic resistance. Eight strains harbored the intrinsic carbapenemase gene *bla_OXA-50_*. The diverse virulence-gene profiles of strains PA2, PA4, and PA6 aligned with their high pathogenicity observed both in vitro and in vivo. However, virulence genotypes sometimes did not correlate with phenotypes, revealing the limitations of relying on static genetic information alone. This study highlights the aquatic environments of the Tibetan Plateau as reservoirs of carbapenem-resistant *P. aeruginosa* with substantial genetic diversity and divergent pathogenic potential, underscoring their public-health relevance.

## 1. Introduction

Carbapenem antibiotics, serving as the “last line of defense” against infections by multidrug-resistant Gram-negative bacteria, are facing a growing resistance crisis that greatly threatens global public health. The prevalence of carbapenem-resistant *Pseudomonas aeruginosa* (*P. aeruginosa*), a major nosocomial pathogen, increases annually, resulting in growing clinical concerns [1]. According to World Health Organization surveillance data, carbapenem-resistant *P. aeruginosa* has been detected in over 20% of cases worldwide [2]. Resistance rates have climbed to 35–50% in affected regions, notably in Southern Europe, South Asia, and Latin America—a trend strongly associated with higher mortality among critically ill patients.

Aquatic environments are major amplifiers and reservoirs in the global antibiotic-resistance crisis. Receiving continuous discharge from medical, agricultural, and domestic sources, various water ecosystems serve as critical hubs for the proliferation, persistence, and dissemination of antibiotic resistance genes (ARGs) and antibiotic-resistant bacteria [3]. Aquatic environments pose public-health risks by allowing antibiotic-resistant pathogens to re-enter human populations via exposure routes such as direct contact, recreation, and the consumption of contaminated irrigated crops, thus forming a clinical–environmental loop that undermines infection control [4,5].

*P. aeruginosa* carrying clinically relevant carbapenemase genes have been detected in various natural and engineered aquatic environments worldwide, including rivers, lakes, wastewater treatment plants, and drinking-water distribution systems [6]. Notably, antibiotic resistance determinants and sequence types (STs) identified in these environmental isolates closely match those observed in clinical settings, strongly suggesting that pathogen exchange occurs between environmental and clinical reservoirs [7]. Furthermore, environmental niches drive substantial diversity in carbapenem-resistant *P. aeruginosa*, yielding novel resistance profiles and strain variants that extend the known resistance landscape beyond clinical settings [8]. Therefore, the sustained surveillance of carbapenem-resistant *P. aeruginosa* in aquatic environments is critical.

The Tibetan Plateau, often called the “Roof of the World,” has an average altitude exceeding 4000 m and exhibits an aquatic environment with extreme conditions and high ecological value [9,10]. Its high-altitude, cold setting, shaped by glacial meltwater, alpine lakes, and rivers, forms a unique system of cold, oligotrophic water bodies [11]. This ecosystem, characterized by hypoxia and intense ultraviolet radiation, hosts aquatic microbial communities with remarkable extremophilic adaptations [12]. However, data on carbapenem-resistant *P. aeruginosa* in the water bodies of the plateau remain considerably scarce.

While environmental–clinical transmission of carbapenem-resistant *P. aeruginosa* has been documented in low-altitude settings, the role of high-altitude pristine aquatic environments as reservoirs remains unknown. We hypothesized that these extreme environments harbor genetically diverse, multidrug-resistant, and potentially pathogenic *P. aeruginosa* strains that may pose a public health risk. To investigate this hypothesis, eight isolates from aquatic environments of the Tibetan Plateau were genomically and phenotypically characterized.

## 2. Materials and Methods

### 2.1. Collection of Water Samples and Bacterial Enrichment

Using sterilized buckets, water samples were collected from glacial meltwater, groundwater, and surface water in Tibet, China, after which they were immediately transported to a laboratory on ice for further analysis. Sampling was conducted between April 2020 and July 2021. To collect microbial biomass, 20 L of water from each sample was filtered through sterile 0.22-μm-pore-size filter membranes. Filters containing retained bacteria were then aseptically cut and placed in Erlenmeyer flasks containing 100 mL of phosphate-buffered saline (PBS). The flasks were then agitated for 20–30 min to elute bacteria from the membranes. After centrifugation of the eluate at 5000× *g* for 10 min, the resulting bacterial pellet was resuspended in 2 mL of PBS.

### 2.2. Isolation and Identification of Carbapenem-Resistant P. aeruginosa

The resulting bacterial suspension was spread onto the CHROMagar^TM^ mSuperCARBA^TM^ agar (CHROMagar, Paris, France) and incubated at 37 °C for 48 h. CHROMagar™ mSuperCARBA™ is a selective chromogenic medium for carbapenem-resistant Gram-negative bacilli; *P. aeruginosa* produces distinctive pink to reddish colonies. Only colonies with this morphology were selected for further analysis. Thereafter, carbapenem-resistant strains were isolated and identified using matrix-assisted laser desorption ionization time-of-flight mass spectrometry (VITEK^®^ MS, bioMérieux version3.2, Marcy-l'Étoile, France) with the VITEK^®^ MS IVD Knowledge Base (version 3.2). Species identification was performed using MALDI-TOF-MS applying a score threshold of ≥2.0 for species-level confidence and ≥1.7 for genus-level confidence. Isolates confirmed via mass spectrometry to be *P. aeruginosa* were defined as carbapenem-resistant *P. aeruginosa* strains.

### 2.3. Antibiotic Susceptibility Testing

Antibiotic susceptibility profiles of *P. aeruginosa* isolates were obtained using the VITEK 2^®^ automated platform (Thermo Fisher, Oxford, UK). *Escherichia coli* ATCC 25922 was used as the quality-control strain, and testing was performed following the manufacturer’s protocol and utilizing a turbidimeter, an AIM automated inoculation system, and an automated susceptibility-testing analyzer (Thermo Fisher Scientific). Minimum inhibitory concentrations were interpreted according to the breakpoints established in the Clinical and Laboratory Standards Institute M100 document (2024 edition) [13].

### 2.4. Assessment of Biofilm Formation by P. aeruginosa Isolates

Biofilm formation was assessed using the crystal-violet staining method [14]. In summary, a 200-μL aliquot of the diluted bacterial suspension (10^6^–10^7^ CFU/mL) was added to each well of a sterile 96-well plate and incubated at 37 °C for 24 h. Wells containing only tryptic soy broth served as negative controls. No specific strong biofilm-forming positive control was used. After incubation, the wells were washed thrice using PBS, and then fixed with 200 μL of 99% ethanol for 15 min, followed by ethanol removal and air-drying for 2 min. Subsequently, the biofilms were stained with 200 µL of 0.1% crystal violet for 15 min, washed thrice using PBS, and solubilized with 30% acetic acid, before using a microplate reader (Molecular Devices, San Jose, CA, USA) to measure the optical density (OD) at 550 nm. Assays were performed in triplicates. The biofilm-forming capacity was classified based on OD550 compared to the *E. coli* ATCC 25922 control (ODc): non-former (OD ≤ ODc), weak (ODc < OD ≤ 2 × ODc), moderate (2 × ODc < OD ≤ 4 × ODc), and strong (OD > 4 × ODc).

### 2.5. Assessment of Invasion Capacity in P. aeruginosa Isolates

Overnight bacterial cultures were subcultured into a fresh medium and grown for 3 h before use. Using the prepared bacterial suspensions, monolayers of HeLa and Caco-2 cells (1 × 10^5^/well; 24-well plate) were infected at a Multiplicity of Infection of 100. After 2 h infection, extracellular bacteria were eliminated by subjecting the cells to three washes with PBS containing 100-μg/mL gentamicin. The cells were then lysed in a PBS buffer supplemented with 0.1% sodium dodecyl sulfate and 1% Triton X-100, and the resulting lysates were serially diluted, plated on LB agar, and incubated for enumeration of viable intracellular bacteria. The invasion rate was calculated as the ratio of intracellular bacteria (2 h post-infection) to the total bacteria in the inoculum. Experiments were conducted in triplicates.

### 2.6. Virulence of P. aeruginosa in Galleria mellonella (G. mellonella) Larvae

*P. aeruginosa* isolates were cultured overnight in Mueller–Hinton broth at 37 °C and subsequently diluted in PBS to a final concentration of 1 × 10^7^ CFU/mL. *G. mellonella* larvae of comparable sizes, weights, and health statuses were randomly assigned to groups of ten. Using a microsyringe, a 10-μL bacterial suspension was administered into the hemocoel of the *G. mellonella* larvae via the left proleg, while control groups received an equal volume of PBS. Post-injection, the *G. mellonella* larvae were incubated at 37 °C in the dark, and survival was recorded every 24 h for 168 h before plotting survival curves [15,16].

### 2.7. DNA Extraction and Whole-Genome Sequencing

Following the manufacturer’s instructions, the Omega Bacterial DNA Kit (D3350-01, Omega Bio-tek, Norcross, GA, USA) was used to extract genomic DNA from *P. aeruginosa* isolates. DNA samples with confirmed purity (A260/A280 ratio of 1.8–2.0) were subjected to Illumina HiSeq 2500 sequencing at Novogene Co., Ltd. (Beijing, China).

To obtain clean data, raw sequencing reads were filtered by removing those containing more than 40% of bases with quality scores ≤ 20, over 10% of N bases, or detectable adapter sequences (allowing ≤ 3 mismatches within a 15 bp overlap). Following the de novo assembly of clean data with SOAPdenovo (v2.04) [17], SPAdes (V4.2.0) [18] and ABySS (2.1.5) [19] across K-mer values (95, 107, and 119), the assembly that generated the least scaffolds was selected. Moreover, contigs from multiple assemblers were merged using the CIS program [20], followed by gap closure via GapCloser (v1.12) to optimize the assembly. Thereafter, contigs shorter than 500 bp were filtered out. Finally, ARGs (e-value ≤ 10^−7^, identity ≥ 96%, coverage ≥ 75%) and virulence genes (e-value ≤ 10^−5^, identity ≥ 96%, coverage ≥ 70%) were predicted using DIAMOND BLASTX (v2.1.6).

### 2.8. Phylogenetic Analysis of P. aeruginosa Isolates

Multilocus sequence typing (MLST) was performed using MLST software v2.32.3 (https://github.com/tseemann/mlst, accessed on 21 April 2026), which uses BLAST (version 2.15.0) to assign STs by aligning the alleles of seven housekeeping genes against the PubMLST database (https://pubmlst.org/, accessed on 21 April 2026). Publicly available *P. aeruginosa* ST profiles were retrieved from the PubMLST database, and combined with the ST data of the eight strains from this study. A minimum spanning tree was then generated using the PHYLOViZ Online platform (https://www.phyloviz.net/, accessed on 21 April 2026).

To assess the genetic relatedness of the *P. aeruginosa* strains, a genome-wide single-nucleotide polymorphism (SNP)-based phylogenetic analysis was conducted using the CSI Phylogeny (1.4) web server (https://cge.food.dtu.dk/services/CSIPhylogeny/, accessed on 21 April 2026). As a reference, the complete genome sequence of *P. aeruginosa* PAO1 (GenBank accession No. NC_002516.2) was used. The analysis included 19 *P. aeruginosa* genomes: eight from the environmental isolates sequenced in this study, and 11 retrieved from the National Center for Biotechnology Information (NCBI) GenBank database. The 11 publicly available genomes were selected based on: (i) geographic diversity (Asia, Europe, and America), (ii) clinical origin (to allow for comparison with our environmental isolates), and (iii) availability of high-quality complete or near-complete genome assemblies. All genome sequences were submitted to the CSI Phylogeny pipeline in FASTA format. Furthermore, to ensure high-quality SNP calling, the analysis was conducted using default parameters, with a minimum depth of 10× at SNP positions, a minimum relative depth of 0.1, a minimum inter-SNP distance of 10 bp, and SNP quality-filtering enabled. A maximum-likelihood phylogenetic tree was subsequently reconstructed from the concatenated high-quality SNP alignment, after which it was exported in Newick format and then visualized and annotated using the Interactive Tree of Life (v6) (https://itol.embl.de/, accessed on 21 April 2026). Bootstrap support values were calculated from 1000 replicates to assess the robustness of the tree topology.

### 2.9. Statistical Analysis

Unless otherwise specified, all quantitative assays were performed in triplicates, and the data were presented as mean ± standard deviation. Moreover, statistical analyses were conducted using GraphPad Prism (version 10.0, GraphPad Software, Boston, MA, USA) and the R software (version 4.3.2, R Foundation for Statistical Computing, Vienna, Austria; http://cran.r-project.org, accessed on 21 April 2026). A *p*-value of < 0.05 was considered statistically significant, with *p* < 0.01 and *p* < 0.001 indicating high significance.

Differences in the biofilm biomass (OD_550_) and invasion rates among the ten *P. aeruginosa* isolates were evaluated using one-way analysis of variance (ANOVA). Although the number of biological replicates per strain was small (*n* = 3), ANOVA is robust to violations of normality when sample sizes are equal and the assumptions of homogeneity of variances are met, as confirmed by Levene‘s test. In the case of a significant difference, as determined using ANOVA, post hoc pairwise comparisons were performed using Tukey’s multiple-comparison test (with letter-coded grouping: a, b, c, and d) in GraphPad Prism. Groups not sharing a common letter differed significantly (*p* < 0.05). Prior to the ANOVA, the homogeneity of variances was assessed using Levene’s test, and no significant deviations were found.

Detailed sequencing quality metrics for all eight isolates (raw read count, clean read count, Q20/Q30 values, genome size, GC content, N50, number of contigs, sequencing depth, and completeness/contamination estimates from CheckM) are provided in Table S4. Genome assembly quality metrics for the eight carbapenem-resistant *P. aeruginosa* isolates are provided in Table S5.

## 3. Results

### 3.1. Phylogenetic Analysis of Carbapenem-Resistant P. aeruginosa Isolates

The results show that even pristine high-altitude aquatic environments harbor genetically diverse, multidrug-resistant, and potentially pathogenic *P. aeruginosa* strains, highlighting an overlooked public-health threat and underscoring the necessity of including remote ecosystems in global One Health surveillance.

Eight carbapenem-resistant *P. aeruginosa* isolates were obtained from the Tibetan Plateau aquatic environments. To investigate the genetic diversity of *P. aeruginosa* isolates, MLST was performed based on the allelic profiles of seven housekeeping genes, namely, *acsA*, *aroE*, *guaA*, *mutL*, *nuoD*, *ppsA*, and *trpE*. Three known STs were identified among the eight isolates (Table S1). ST1420 predominated, comprising five isolates (PA3, PA4, PA5, PA7, and PA8), while ST3229 was represented by two isolates (PA1 and PA6), and ST549 by one isolate (PA2).

**Figure 1:**
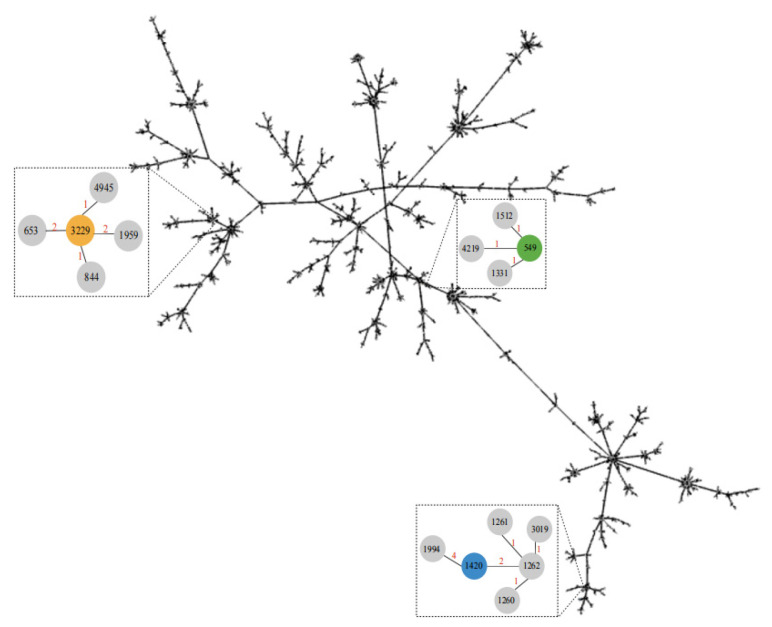
Minimum spanning tree of *P. aeruginosa* isolates based on multilocus sequence typing (MLST).

To elucidate phylogenetic relationships among the identified STs and globally distributed *P. aeruginosa* lineages, a minimum spanning tree was constructed based on concatenated allelic profiles of the representative STs retrieved from the PubMLST database (Figure 1). The dominant ST1420 clustered closely with ST1262 and ST1994, exhibiting two or four allelic differences, indicating close genetic relatedness and possible shared ancestry. ST3229 formed a clade with ST4945 and ST844, differing by only one allele. ST549 clustered with ST1512, ST4219, and ST1331 (one allelic difference each). Thus, the genetic heterogeneity differs substantially among the aquatic *P. aeruginosa* isolates. The close relatedness of these STs to previously reported environmental or clinical isolates (e.g., ST844 and ST1512) raises public-health concerns.

**Figure 2:**
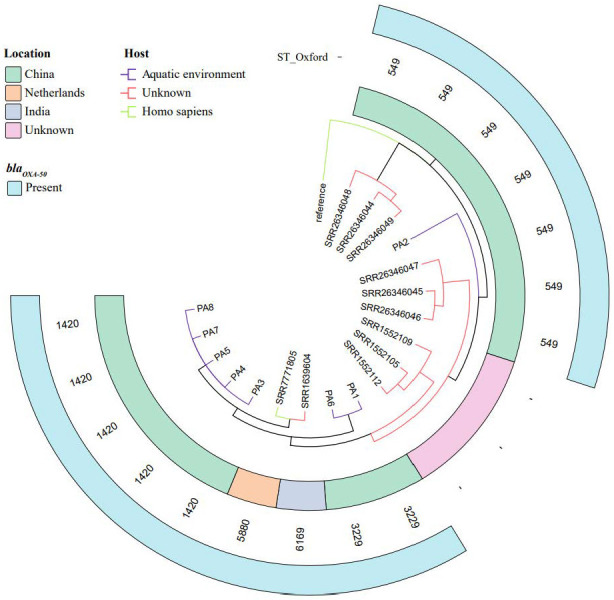
Core-genome SNP-based phylogeny of *P. aeruginosa* isolates, rooted with reference strain *P. aeruginosa* PAO1. The tree delineates clades corresponding to sequence types (STs), geographic origin, host source, and *bla_OXA-50_* carriage.

To resolve the evolutionary relationships among the *P. aeruginosa* isolates assessed in this study as well as in publicly available genomes, a maximum-likelihood phylogeny was reconstructed based on core-genome SNPs, with the *P. aeruginosa* PAO1 reference genome used as an outgroup (Figure 2). The ST1420 isolates PA3, PA4, PA5, PA7, and PA8 clustered together, with all strains sharing identical core-genome sequences. The five ST1420 isolates (PA3, PA4, PA5, PA7, and PA8) exhibited identical core-genome sequences (zero branch length) despite being collected across different seasons (April 2020 to July 2021). This finding suggests the presence of a highly stable clonal lineage (ST1420) in the Tibetan Plateau aquatic environment that persisted over an extended period, rather than a single transient clonal outbreak. In addition, these isolates formed a distinct branch that showed close genetic relatedness to an isolate from India (SRR1639604, ST6169 and *bla_OXA-50_*-positive) and an isolate from the Netherlands (SRR7771805, ST5880 and *bla_OXA-50_*-positive). A clearly distinct branch emerged, comprising ST3229 isolates PA1 and PA6. The phylogenetic tree further revealed close genetic relatedness between environmental isolates from this study and clinical or human-associated isolates from geographically distant regions (e.g., SRR1639604 from the India and SRR7771805 from The Netherlands). Therefore, these environmental *P. aeruginosa* lineages may pose public-health risks, and the results support the hypothesis of global carbapenem-resistant *P. aeruginosa* clone dissemination across environmental and clinical reservoirs.

### 3.2. Characterization of Antibiotic Susceptibility Profiles and Associated Genetic Determinants in P. aeruginosa Isolates

**Figure 3:**
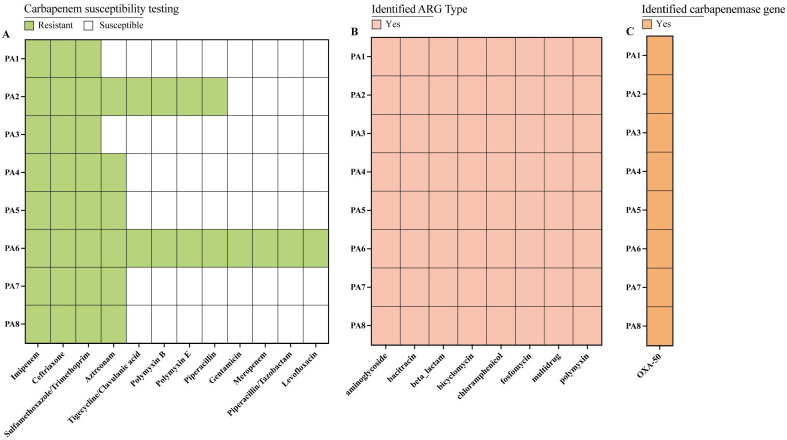
Antimicrobial susceptibility profiles and genetic determinants in *P. aeruginosa* isolates. (**A**) Heatmap of resistance phenotypes of eight carbapenem-resistant *P. aeruginosa* isolates against 12 antibiotics. (**B**) Distribution of resistance genes among the isolates. (**C**) Carriage of *bla_OXA-50_* in the eight isolates.

All eight isolates were multidrug-resistant, exhibiting 100% resistance to imipenem, ceftriaxone, and trimethoprim–sulfamethoxazole, along with 75% resistance to aztreonam, as determined through antibiotic susceptibility testing. Additionally, 25% of isolates were resistant to tigecycline/clavulanic acid, polymyxin B, polymyxin E, and piperacillin and 12.5% to the remaining antibiotics tested (Figure 3A). Strain PA6 was resistant to 12 antibiotics, whereas strain PA2 showed a comparatively lower resistance rate of two-thirds (66.67%). The multidrug resistance observed in environmental isolate PA6 highlights the urgency for enhanced surveillance under the One Health framework. The findings reveal a concerning situation, as the clinical management of *P. aeruginosa* infections relies on first-line core agents such as imipenem, meropenem, piperacillin, and piperacillin/tazobactam.

Resistance-gene annotation identified 40 ARGs among the eight *P. aeruginosa* isolates, conferring resistance to eight major antibiotic classes (Table S2). Moreover, the carbapenem resistance phenotype found in all eight carbapenem-resistant *P. aeruginosa* strains may be attributed to the presence of *bla_OXA-50_* (Figure 3C). All strains were resistant to ceftriaxone, consistent with the intrinsic resistance of *P. aeruginosa* to this drug—attributed to its instability against the species’ resident AmpC β-lactamase. In the PA6 strain, the aminoglycoside resistance gene *APH(3′)-IIb* was annotated and found to be consistent with its phenotypic resistance to aminoglycosides such as gentamicin (Table S2) [21]. Likewise, the presence of polymyxin resistance genes was aligned with the observed resistance to both polymyxin B and E. Pseudomonas-derived cephalosporinase (PDC) is a chromosomally mediated AmpC β-lactamase produced by *P. aeruginosa*, and its overexpression, primarily driven by mutations in regulatory genes, represents a principal mechanism underlying aztreonam resistance [22]. However, strains PA1 and PA3, annotated with the *bla_PDC-5_* gene, remained susceptible to aztreonam. PDC-mediated aztreonam resistance typically requires mutations in regulatory genes (e.g., ampD or ampR) to drive enzyme overexpression. PA1 (ST3229) and PA3 (ST1420) remained susceptible to aztreonam, possibly due to the absence of such inducing mutations. This discrepancy underscores the inherent limitation of static genomic annotation in predicting resistance phenotypes; specifically, gene carriage does not guarantee functional resistance.

### 3.3. Virulence Phenotype of P. aeruginosa Isolates In Vitro

**Figure 4:**
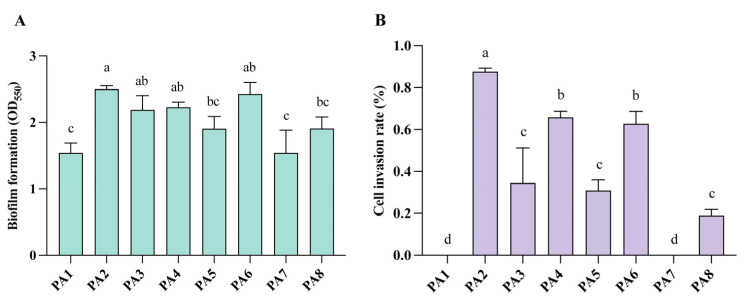
In vitro virulence profiles of carbapenem-resistant *P. aeruginosa* isolates. (**A**) Biofilm-forming capacity (*n* = 3). (**B**) Invasive capacity (*n* = 3). Different letters (a, b, c, and d) indicate statistically significant differences between groups (*p* < 0.05, Tukey’s multiple-comparisons test).

Biofilm formation is an important feature of bacterial virulence, playing a major role in evading host immune defenses and enhancing antibiotic resistance [23]. Therefore, this study assessed the biofilm-forming capacity of the *P. aeruginosa* isolates. The biofilm-forming capacity was classified by comparing the OD of each isolate with that of the control *E. coli* ATCC 25922 (ODc), as follows: non-former, OD ≤ ODc; weak, ODc < OD ≤ 2 × ODc; moderate, 2 × ODc < OD ≤ 4 × ODc; and strong, OD > 4 × ODc. Strains PA2, PA3, PA4, and PA6 exhibited moderate biofilm-forming capacity, while the rest showed weak capacity (Figure 4A). Notably, strains PA2 and PA6 showed resistance to multiple antibiotics, consistent with their relatively high biofilm-forming ability.

The invasiveness of *P. aeruginosa* isolates was assessed using HeLa and Caco-2 cells. Strains PA2, PA4, and PA6 exhibited relatively high invasiveness (0.628–0.876%), whereas PA3, PA5, and PA8 showed moderate levels (0.190–0.344%). Strain PA1 and PA7 lacked invasiveness (Figure 4B).

Strains PA2, PA4, and PA6 exhibited both moderate biofilm formation and relatively high invasive capacity. The collective phenotypic superiority of these strains in biofilm formation and cellular invasion corroborates their enhanced pathogenic potential.

### 3.4. Virulence Phenotype of P. aeruginosa Isolates In Vivo

**Figure 5:**
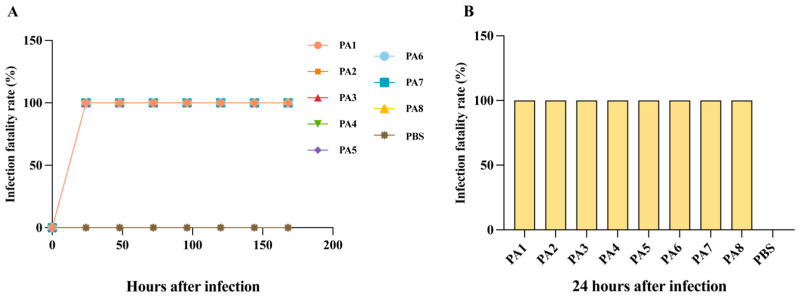
Lethality of *G. mellonella* larvae infected with carbapenem-resistant *P. aeruginosa* isolates. (**A**) Time-dependent lethality curves grouped by individual isolate over time (*n* = 10). (**B**) Lethality at 24 h post-infection by isolate.

This study used the *G. mellonella* larval model to assess the in vivo virulence of the *P. aeruginosa* strains. *G. mellonella* larvae infected with all eight tested isolates showed complete melanization and mortality within 24 h post-infection (in Figure 5A), while no mortality was observed in the PBS control group. The in vivo pathogenic phenotypes of strains PA2, PA4, and PA6 closely mirrored their in vitro profiles. Within 24 h post-infection, strains PA2, PA4, and PA6 achieved 100% lethality in *G. mellonella* larvae.

### 3.5. Analysis of Virulence Genes in P. aeruginosa Isolates

**Figure 6:**
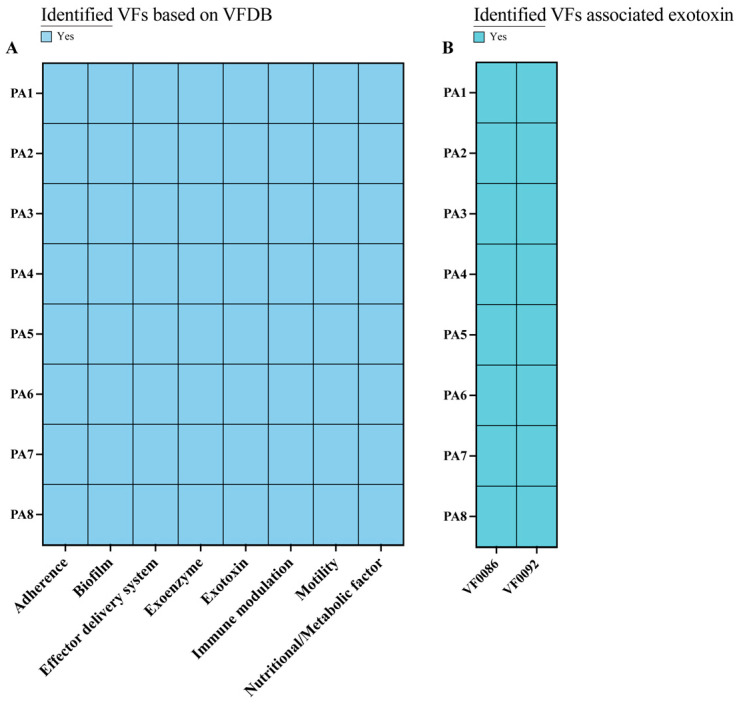
Distribution of virulence factors in *P. aeruginosa* isolates. (**A**) Virulence-gene categories based on the VFDB classification. (**B**) Presence of exotoxin-associated virulence factors VF0086 (ExoS) and VF0092 (ExoT).

Based on the Virulence Factor Database classification, the virulence genes of the eight *P. aeruginosa* isolates were identified, and they were affiliated with eight categories, namely, adherence, biofilm formation, an effector delivery system, an exoenzyme, an exotoxin, immune modulation, motility and nutritional/metabolic factor (Figure 6A). Among the eight isolates, all carried genes related to effector delivery systems, making it the predominant category and accounting for the majority of all virulence genes identified (Table S3).

The exotoxin-associated virulence factors VF0086 and VF0092 were detected in all eight isolates (Figure 6B). VF0086 (ExoS) and VF0092 (ExoT) are effector proteins secreted via the type-III secretion system [24,25]. ExoS is a bifunctional toxin that performs ADP-ribosyltransferase and GTPase-activating protein (GAP) activities, leading to acute cytotoxicity via a disruption of host–cell signaling and the cytoskeleton, thereby promoting tissue invasion. ExoT also exhibits GAP activity (and weak ADP-ribosyltransferase activity), mainly inhibiting host–cell migration, phagocytosis, and wound healing, thereby promoting bacterial persistence and chronic infection. The presence of both ExoS and ExoT in *P. aeruginosa* is well documented, with both playing key roles in mediating its pathogenicity [26,27,28].

In strains PA2, PA4, and PA6, the robust in vitro and in vivo virulence was aligned with their comprehensive virulence-gene profiles. These strains possessed a diverse functional repertoire of virulence genes, including adherence, biofilm formation, effector delivery systems, exoenzymes, exotoxins, immune modulation, motility, and nutritional/metabolic factors. Consistent with their genotype, these strains exhibited moderate biofilms, high invasiveness, and 100% larval mortality, confirming a strong link between genetic potential and functional output [29,30]. However, the virulence genotype–phenotype correlation was not strictly linear, revealing the inherent limitations of static genomic data in predicting complex pathogenic traits.

## 4. Discussion

The occurrence and characterization of carbapenem-resistant *P. aeruginosa* in the Tibetan Plateau’s aquatic environments remain elusive. This study systematically characterized eight carbapenem-resistant *P. aeruginosa* isolates from aquatic systems on the Tibetan Plateau and revealed their genetic diversity and phylogeny, resistance and virulence traits, and associated genetic determinants in this high-altitude extreme environment. The findings are of the utmost importance for mitigating the health risks associated with carbapenem-resistant *P. aeruginosa* in the aquatic environments of the Tibetan Plateau.

MLST analysis revealed three distinct STs among the eight carbapenem-resistant *P. aeruginosa* isolates, with ST1420 being the predominant clone (62.5%, 5/8), a sequence type first recorded from a clinical isolate in China and documented in the PubMLST global database, suggesting its potential for cross-habitat dissemination between clinical and environmental reservoirs. Moreover, this result suggests its potential for cross-habitat dissemination. Analysis of the PubMLST database further supported this notion: ST1262, closely related to ST1420, was isolated from a water sample in Spain in 2010, suggesting a potential environmental reservoir. ST844 and ST653, both single-locus variants of ST3229, have been recovered from clinical specimens including sputum, bronchial lavage, and urine across Australia and China, indicating their clinical relevance and wide geographic distribution. Notably, several environmental isolates in this study (e.g., PA3, PA4, PA5, PA7, and PA8) were genomically proximate to a human-associated strain of ST5880 from the Netherlands. These findings suggest a possible genetic relatedness between environmental carbapenem-resistant *P. aeruginosa* reservoirs and clinical infections. However, we acknowledge that without temporally and geographically matched clinical samples, direct transmission cannot be proven. The observed relatedness may reflect shared ancestry or independent acquisition of similar genetic traits rather than recent cross-habitat transmission. Together, these findings support the hypothesis that the STs identified in this study are not confined to the Tibetan Plateau but are part of globally disseminated lineages with both environmental and clinical significance. They provide compelling genetic evidence for potential epidemiological links between environmental carbapenem-resistant *P. aeruginosa* reservoirs and clinical infections and underscore the urgency to incorporate systematic environmental surveillance into the “One Health” framework.

All eight *P. aeruginosa* isolates were found to be multidrug-resistant, showing 100% resistance to imipenem, ceftriaxone, and trimethoprim–sulfamethoxazole, and 75% to aztreonam. Notably, strain PA6 exhibited complete resistance to all 12 tested antibiotics, meeting the criteria for extensively drug-resistant (XDR) *P. aeruginosa*, an alarming finding in pristine environmental waters. All eight carbapenem-resistant *P. aeruginosa* isolates carried the intrinsic *bla_OXA-50_* gene, which aligns with their carbapenem resistance phenotypes.

The presence of multidrug-resistant *P. aeruginosa* in pristine high-altitude aquatic environments raises questions about potential sources of dispersal. We propose three possible explanations: (i) atmospheric deposition of dust carrying resistant bacteria from distant anthropogenic sources, as ARGs have been detected in free-troposphere aerosols at remote high-mountain sites [31]; (ii) wildlife (e.g., migratory birds) that can transport resistant bacteria over long distances, as evidenced by studies around Qinghai Lake, an ecologically similar high-altitude environment in China, where migratory birds were identified as a major source of environmental antibiotic resistance deposited into water bodies via fecal contamination [32]; and (iii) anthropogenic activities including tourism and research expeditions on the plateau, given that a clear link between human activity intensity and elevated antibiotic resistance levels has been demonstrated in remote glacier and lake water samples [33].

In current practice, first-line therapy for *P. aeruginosa* primarily involves the use of carbapenems (imipenem and meropenem) and piperacillin/tazobactam, or piperacillin alone. Notably, carbapenem-resistant *P. aeruginosa* isolates from the Tibetan Plateau’s aquatic environments were highly resistant (75–100%) to imipenem, ceftriaxone, trimethoprim–sulfamethoxazole, and aztreonam. Furthermore, strain PA6 displayed resistance to meropenem, piperacillin/tazobactam, polymyxin B/E, and gentamicin. This finding is of particular concern, as these antibiotics are still critically important for treating *P. aeruginosa* infections—especially those involving multidrug-resistant strains—in clinical practice beyond the plateau region.

In vivo assays showed that all eight carbapenem-resistant *P. aeruginosa* isolates caused 100% mortality in *G. mellonella* larvae within 24 h, indicating the retention of substantial pathogenic potential in an invertebrate host. However, the strains exhibited marked heterogeneity in terms of biofilm formation and epithelial cell invasion. In addition, no strong positive control was used in the biofilm assay; only the weak biofilm-forming *E. coli* ATCC 25922 was used as a reference control for relative classification. This may affect the absolute assessment of biofilm-forming capacity of the isolates.

This study offers the first multidimensional characterization of carbapenem-resistant *P. aeruginosa* isolates from aquatic environments of the Tibetan Plateau, providing crucial insights into the occurrence and potential the public-health risks of clinically relevant resistant pathogens in pristine ecosystems. We acknowledge that without temporally and geographically matched clinical samples, direct transmission from Tibetan Plateau waters to human infections cannot be proven. The observed phylogenetic relatedness may reflect shared ancestry or independent acquisition of similar genetic traits rather than recent cross-habitat transmission. Nevertheless, the existing literature supports a bidirectional human-environment exchange of *P. aeruginosa*, with recreational water contact documented as a route of human infection, and hospital effluent demonstrated as a route by which resistant clinical clones enter environmental waters [34,35,36,37]. The Tibetan Plateau is increasingly frequented by tourists and researchers who may come into direct contact with these water bodies, representing a plausible route of human exposure [38,39,40]. The phylogenetic relatedness observed in this study underscores the urgency of integrating remote aquatic environments into systematic One Health surveillance frameworks.

Another limitation is the use of a selective medium (CHROMagar™ mSuperCARBA™) for isolation, which specifically enriches for carbapenem-resistant bacteria. Therefore, the prevalence of carbapenem-susceptible *P. aeruginosa* in these aquatic environments could not be determined, and our study does not capture the full diversity of the resident *P. aeruginosa* population. We acknowledge that without temporally and geographically matched clinical samples, direct transmission from Tibetan Plateau waters to human infections cannot be proven. Nevertheless, the phylogenetic relatedness observed in this study suggests the potential for environmental reservoirs to contribute to the dissemination of clinically relevant resistant lineages.

## 5. Conclusions

This study provides the first comprehensive characterization of carbapenem-resistant *P. aeruginosa* isolated from the Tibetan Plateau’s aquatic environments. The eight studied carbapenem-resistant *P. aeruginosa* isolates displayed substantial genetic diversity, comprising three STs (ST1420, ST3229, and ST549). Thus, the results revealed that even pristine high-altitude aquatic ecosystems harbor phylogenetically diverse resistant lineages. The close genomic relatedness between these environmental isolates and geographically distant clinical strains underscores the need to integrate environmental monitoring into One Health frameworks. Extreme resistance was observed, including in the multi-resistant strain PA6 (resistant to 12 antibiotics, including polymyxins), confirming that such traits also exist in pristine environments. In all eight strains, carbapenem resistance was primarily mediated by *bla_OXA-50_*, underscoring the role of this intrinsic gene in environmental settings. Strains PA2, PA4, and PA6 demonstrated high pathogenicity in vitro and in vivo, corroborated by their diverse virulence-gene profiles. These findings highlight the urgency to include remote and extreme environments in global antibiotic resistance monitoring under the One Health framework to better understand the ecology, evolution, and public-health implications of environmental carbapenem-resistant *P. aeruginosa*.

## Data Availability

The original contributions presented in this study are included in the article/Supplementary Material. Further inquiries can be directed to the corresponding authors.

## Supplementary Materials

The following supporting information can be downloaded at: https://www.mdpi.com/article/10.3390/microorganisms14051094/s1, Table S1: MLST profiles and isolation sources of *Pseudomonas aeruginosa* isolates; Table S2: Resistance gene profiles of the 10 carbapenem-resistant *Pseudomonas aeruginosa* isolates; Table S3: Virulence factor profiles of the 10 carbapenem-resistant *Pseudomonas aeruginosa* isolates; Table S4: Genome assembly quality metrics for the eight carbapenem-resistant *P. aeruginosa* isolates in this study; Table S5: Epidemiological information of closely related sequence types (STs) to the STs identified in this study, retrieved from the PubMLST database.
